# Nodular Regenerative Hyperplasia and Portal Hypertension in a Patient with Coeliac Disease

**DOI:** 10.1155/2011/938580

**Published:** 2011-08-28

**Authors:** Erwin Biecker, Hans-Peter Fischer, Michael Schepke

**Affiliations:** ^1^Department of Gastroenterology and Hepatology, HELIOS Klinikum Siegburg, Ringstraße 49, 53721 Siegburg, Germany; ^2^Department of Pathology, University Hospital Bonn, Sigmund-Freud Straße 25, 53105 Bonn, Germany

## Abstract

Nodular regenerative hyperplasia (NRH) of the liver is often associated with rheumatologic or lymphoproliferative disorders and a cause of portal hypertension in some patients. We report the case of a 71-year-old patient with celiac disease and unexplained portal hypertension. Biopsy of the liver revealed NRH as the underlying cause. The patient did not suffer from an autoimmune, rheumatologic or lymphoproliferative disease. A thrombophilic disorder that might cause NRH was ruled out. Celiac disease is often associated with mild elevation of liver enzymes and steatosis of the liver, but the association with NRH was described in only a few patients. We discuss the possible relationship of celiac disease and NRH.

## 1. Introduction

Nodular regenerative hyperplasia (NRH) of the liver is histologically characterized by an atrophy of the hepatocytes in the central areas and compensatory proliferation of the hepatocytes in portal regions. The occlusion of small portal vein branches is considered to be the basic lesion; however, the exact pathogenesis is not known [1]. Nodular regenerative hyperplasia is frequently associated with underlying lymphoproliferative or rheumatological disorders; an association with drugs like azathioprine has also been described [1]. Liver function is usually well preserved in patients with NRH, but portal hypertension might be present at least in some of the affected patients.

Gluten-sensitive enteropathy is associated with mild liver abnormalities like hypertransaminasemia in up to 40% of patients [3], but more severe involvement of the liver is rare and usually resolves on a gluten-free diet [4]. 

We describe a patient with long-standing, so far unrecognised gluten-sensitive enteropathy, NRH, and severe portal hypertension. The possible causes for the relationship between NRH and gluten-sensitive enteropathy are discussed.

## 2. Case Report

The 71-year-old male patient was admitted to our hospital for further diagnostic workup of microcytic anemia. The patient complained of fatigue during the last months but denied any signs of gastrointestinal bleeding. He did not report weight loss, nor did he complain of any gastrointestinal symptoms like diarrhoea or steatorrhea.

Physical examination showed an anemic but well nourished patient in good health. The spleen was not palpable. The laboratory workup revealed moderate microcytic anemia (Hb 9.0 g/dL, MCV 71 fL) and a low ferritin (8 ng/mL, normal range 30–400 ng/mL). The transaminases were mildly elevated (GOT 50 U/L, GPT 51 U/L, normal range 9–38 U/L, and 10–41 U/L, resp.). Bilirubin, *γ*GT as well as the prothrombin time were within normal ranges. Tests for hepatitis B and C and autoantibody tests (antinuclear, antineutrophil cytoplasmatic, antimitochondrial, antismooth muscle) were all negative. 

Liver ultrasound as well as liver NMR revealed mild fibrosis/steatosis, but no signs of cirrhosis. The portal, splenic and the superior mesenteric veins were all dilated but patent. A recanalised umbilical vein was present. Spleen-size was in the normal range. 

Upper endoscopy revealed esophageal varices grade III with cherry red spots but no signs of recent bleeding. Because of the anemia, a duodenal biopsy was taken and showed short villi along with epithelial cell damage and a dense lymphoplasmacellular infiltrate of the lamina propria. The findings were diagnostic for celiac sprue. In addition, IgA antitransglutaminase antibodies were highly positive (82 U/mL, normal range <5 U/mL).

**Figure 1:**
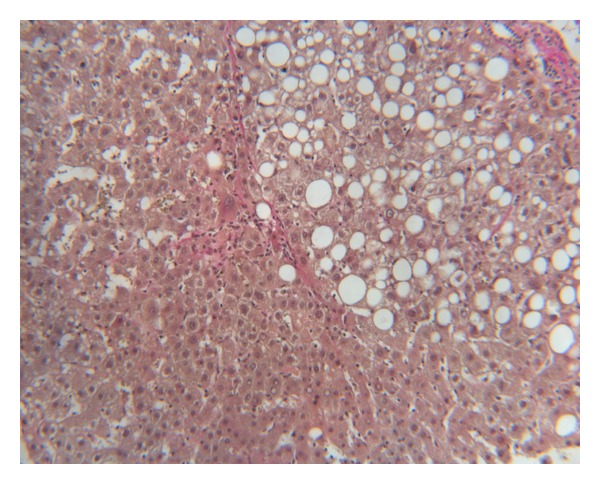
Initial nodular regenerative hyperplasia of the liver with localised fatty degeneration.

For further workup of the unexplained portal hypertension and the mildly elevated transaminases, a percutaneous liver biopsy was done. Histology showed steatosis of the liver and the typical findings of nodular regenerative hyperplasia (Figure 1). In addition, a thrombophilia screen was performed but revealed no abnormalities (assays for proteins C and S, antithrombin III, IgA, IgG, IgM anticardiolipin antibodies (aCL), Factor V Leiden, prothrombin-G20210A-mutation, homocysteine levels were all negative). 

The patient was put on a gluten-free diet and medical treatment with propranolol for primary prophylaxis of bleeding from esophageal varices was started. Six months later, the haemoglobin level and the transaminases were in the normal range.

## 3. Discussion

We describe a patient with portal hypertension, mild liver abnormalities, and anemia caused by gluten-sensitive enteropathy and NRH. 

Portal hypertension and its complications like esophageal varices and ascites are often present in patients with NRH [1, 2]. The liver function is generally preserved and liver functions test are normal or only slightly abnormal. In a high percentage of affected patients, the diagnosis of NRH is missed on imaging procedures like ultrasound, computed tomography or magnetic resonance imaging [5–7]. A liver biopsy is mandatory for diagnosis, but even on a routine needle biopsy, the diagnosis is missed in some patients [8].

Nodular regenerative hyperplasia of the liver is characterized by nodules in varying sizes which replace the normal liver parenchyma. On histology, NRH is distinguished from cirrhosis of the liver by the absence of fibrosis, the separation of nodules of regenerating hepatocytes by atrophic parenchyma and the curvilinear compression of the central veins by regenerating nodules [9]. 

The most accepted pathophysiologic concept for the development of NRH is the diminished perfusion of small branches of the portal venous system. The decreased blood flow causes atrophy of the hepatocytes in central areas that is compensated by hepatocyte proliferation in portal regions [1]. However, the exact pathomechanism is not known. 

Nodular regenerative hyperplasia is a disease that affects mainly patients who are 50 years or older. The disease is often associated with rheumatologic or lymphoproliferative disorders. In addition, autoimmune disorders like Sjögren's syndrome, autoimmune thyroid disease, and rheumatoid arthritis [10, 11] are associated with celiac sprue. The incidence of autoimmune disorders is significantly higher in patients with longstanding, untreated, or poorly controlled coeliac disease [12]. Familial forms or an association with the Budd-Chiari-syndrome have also been described [13, 14]. An association with drugs like thioguanine, busulfan, and azathioprine is also known. 

Furthermore, a number of thrombophilic disorders like polycythemia vera, protein S deficiency [15], and IgG antiphospholipid syndrome [16] are associated with NRH. To our best knowledge, there are only a few patients with NRH and concomitant coeliac disease reported in the literature [17–19]. Austin and coworkers described two patients with refractory celiac disease and NRH who were positive for IgA anticardiolipin antibodies (aCL) [17]. The authors postulated that gluten-specific T cells drive an IgA autoantibody response to both transglutaminase and protein/phospholipid complexes leading to the formation of IgA aCL, causing high local IgA aCL concentrations in the portal vein of these patients. This high local concentration of IgA aCL is considered to increase the risk of thrombus formation in small branches of the portal vein. More than 10% of patients with celiac disease are positive for IgG or IgM aCL [20]. If the presence of aCL is the link (or the cause) between coeliac disease and NRH, one would suspect a higher prevalence of systemic thrombosis and/or NRH in patients with celiac disease. Recently, we reported a young patient with celiac disease, NRH, and portal hypertension who was, like the actual patient, negative for IgA aCL antibodies [19]. In contrast to the actual patient, who was newly diagnosed and was therefore not on a gluten-free diet, the previously reported young patient was on a gluten-free diet for a long time and responded well to the diet. Since both of our patients had no IgA aCL antibodies they differed widely with respect to age, disease duration, and gluten-free diet, the proposed association between these factors and IgA aCL antibodies does appear unlikely.

In conclusion, we can only speculate on the pathophysiological link between NRH and celiac disease in our patient. Since we were not able to detect IgA aCL antibodies in both of our patients, we think that—at least in some patients with celiac disease—IgA aCL antibodies in the portal system are not the pathophysiological basis of NRH in patients with gluten sensitive enteropathy.
